# How Useful? Fish-Friendly Irrigation Guidelines for the Lower Mekong Lack Definition in Five Key Areas

**DOI:** 10.1007/s00267-023-01855-4

**Published:** 2023-07-15

**Authors:** Nicolette Duncan, Jennifer Bond, John Conallin, Lee J. Baumgartner

**Affiliations:** Gulbali Institute for Agriculture, Water and Environment, Charles Sturt University, P.O Box 789, Albury, NSW 2640 Australia

**Keywords:** Fishway guidelines, Fish passage, Multifunctional irrigation, Integrated water management, Guideline evaluation

## Abstract

A proliferation of irrigation infrastructure throughout the Mekong River has impacted the ability of certain fish species to migrate to fulfil their lifecycle. In response, fishways, a type of fish-friendly irrigation structure, have been developed to provide passage for these fish. In recent years, several guidelines documents providing guidance on fish-friendly irrigation structures and their construction have been published. The development process from guideline inception to publication is unclear, while their purpose, audience, and contribution to fishway practice are vague. This study is the first to review the development of three fish-friendly guideline documents, using structural criteria analysis, combined with qualitative data from 27 key informant interviews. It aimed to understand document elements such as purpose, audience, scope, and framing. The results showed reviewed guideline utility and impact could be improved by attention to five key aspects, namely: definition of target audience; engagement of target audience in guideline design; definition of guideline scope; specificity of recommendations; and evaluation. Attention to these 5 aspects may result in guidelines that are perceived as more useful by their target audience and have greater impact on water management practice.

## Introduction

The Lower Mekong Basin (LMB) is the world’s most productive inland fishery (Baran et al. 2007) and fish are a dietary staple throughout the region, defined as parts of Thailand, Laos, Cambodia, and Vietnam (Lynch et al. 2019). Indeed fish often represent the most available and accessible animal protein source for approximately 60 million people, or 60% of LMB residents (Vu et al. 2021).

Aquatic habitat connectivity shapes Mekong fishery production by influencing water quality, fish habitat and fish migration (Conallin et al. 2019; McCartney et al. 2019; Vu et al. 2021). Thousands of water control structures such as dams, weirs, and barrages, fragment the LMB river-floodplain ecosystem, mainly to support rice agriculture (Lynch et al. 2019). Declines in LMB fisheries have been observed due to habitat fragmentation (Baran and Myschowoda 2009; Yoshida et al. 2020; Vu et al. 2021), amongst other factors. Addressing habitat connectivity can support LMB government, regional and global health and livelihood improvement goals by maintaining or enhancing fisheries as a food resource (Dubois et al. 2019; McCartney et al. 2019). To recentre positive development outcomes for Mekong communities, actors such as the United Nations in their Sustainable Development Goals (SDGs – United Nations 2015) call for holistic management of water resources via Integrated Water Management (IWM - Bouwer 2000), underpinned by adaptive management (Baumgartner et al. 2021a; Newson 2008).

Fish-friendly irrigation structures sit within IWM and refer to a suite of innovations which, through water infrastructure design and operation, support fisheries. These include adaptations to traditional water control infrastructure as well as recognising opportunities for fish habitat provision within agricultural landscapes. Fishways are one such innovation which has been tested in the LMB to provide passage for fish through or around water control infrastructure (Baumgartner et al. 2014). Fishways function by reducing into increments the height that migrating fish must climb over weirs, through creation of a series of stepped pools which mimic naturally graduated upstream migratory routes. Fishways can be more or less naturalistic, on a spectrum between specifically constructed rock ramps to cast concrete vertical slot and cone designs, as well as fish locks and lifts (ADB 2020).

Applied experience of fishways has been mixed (Baumann and Stevanella 2012; Baumgartner et al. 2016; Silva et al. 2018; Theparoonrat 2021), generally due to incompatibility between fishway design and the swimming abilities of local fish species (Mallen‐Cooper and Brand 2007). To guide water managers in implementation, fish-friendly irrigation guidelines (FFIGs) have been developed by influential LMB stakeholders, namely: the Asian Development Bank’s *Diversion weirs and fish passages for small-scale irrigation: Lao People’s Democratic Republic* (ADB 2020); the Food and Agriculture Organisation of the United Nations (FAO) *An ecosystem approach to promote the integration and coexistence of fisheries within irrigation systems* (Gregory et al. 2018); and FAO in collaboration with WorldFish and International Water Management Institute’s *Increasing the benefits and sustainability of irrigation through the integration of fisheries: A guide for water planners, managers and engineers* (FAO et al. 2020). Further, the Mekong River Commission (MRC) is anticipated to publish a revision of their guideline (Marsden et al. 2014) in 2023, representing the fourth FFIG published for the LMB in five years.

The current study aims to compare and review Gregory et al. (2018), ADB (2020), and FAO et al. (2020) to establish similarities, dissonances, and areas for improvement, by engaging a recently published framework for guideline development (Nieuwlaat et al. 2021). The current study also aims to illuminate the development process that conceived these three guidelines and comment on their role from LMB irrigation and water management practitioners’ perspectives.

## Literature Review

Holistic water management and fishway implementation requires water managers to navigate complex and evolving best practise advice (Thorncraft et al. 2006; Baumgartner et al. 2018). Guidelines have a long history in water management, commonly focussing on singular management aspects such as IWM (e.g. Besseling 2006); participatory management (Krywkow 2007); and irrigation efficiency improvements (Phengphaengsy and Hiroshi 2006). It is worthwhile interrogating how the documents could be improved for greater impact and utility (Karppinen and Moe 2012).

GLs are a ubiquitous communication tool (Kaiser et al. 2022; Valentine 2017), often constituted of expert consensus reports of best practise (Shaneyfelt and Centor 2009). They aim to provide standardised, evidence-based advice by which decision-makers can determine the course and nature of action (Shen et al. 2021). Various guideline attributes including recency (del Mar Seguí et al. 2011) and scope (O’Connell et al. 2016) can influence guideline impact on practice. Shaneyfelt and Centor (2009) lament that often guidelines represent strongly biased opinion-based marketing tools. Whether or not it’s intentional, interpretive differences and assumptions likely contribute to framing guideline content (O’Connell et al. 2016).

Shekelle et al. (1999) categorise the process of creating a guideline into five main tasks, namely: 1. Defining the subject area and scope; 2. Convening development workshops; 3. Systematically reviewing the evidence; 4. Translating evidence into recommendations; and 5. External review. Employing such a structured approach has benefits such as reducing strategic drift (Sammut-Bonnici 2014), helping to guard against bias (Shaneyfelt and Centor 2009) and ensuring guideline rigor through robust discussion and review (Tsopra et al. 2018).

Development rigour and presentation clarity (Cluzeau et al. 1999) have been found to be useful indicators of guideline utility (Callender, 2018) however guidelines are frequently criticised for lacking structural clarity (Cochrane et al. 2007; Salbach et al. 2020). Many organisations lack formal procedures to review or update their guidelines (Shaneyfelt and Centor 2009). For water management, a lack of evaluation is problematic on two fronts. Firstly, published guidelines may be underutilised due to low compatibility with the operational reality into which they are deployed. Misalignment between guideline recommendations and practical reality was found to be a primary cause of underutilisation, even for prominent water management guidelines such as the World Commission on Dams Report (Nakayama and Fujikura 2006). Secondly, without evaluation, lessons from users’ experience with published guidelines may not be incorporated into an adaptive management processes which may improve them. Underpinning this are findings by Shiffman et al. (2005) that show recommendations that fail *executability* criteria (i.e. do not clearly communicate what to do) or *decidability* criteria (i.e. when to do it), are not ready for implementation. The perceived strength of recommendations (i.e. are guidelines necessitating or suggesting action – Lomotan et al. 2010) is another variable which may influence the extent to which guideline advice is acted upon. Finally, externalities such as time, and knowledge, human, economic and technical resources can also influence guideline impact (Fulcher and Gosselin-Acomb 2007).

Nieuwlaat et al. (2021) developed a framework to assess guideline structure, aiming to support development of guidelines complete with components that allow users to locate critical information easily. Most examples of other guideline evaluation tools in the literature are overwhelmingly from the health sector, focusing on guideline structure (e.g. Cluzeau et al. 1997; Cluzeau 2003; Brouwers et al. 2010) and recommendations (e.g. Shiffman et al. 2005). Methods range from algorithmic coding of guideline language for statistical analysis, noted as being time consuming and dependent on the guideline’s original sentence structure (Tsopra et al. 2018), to expert elicitation and interviews (Cluzeau et al. 1999).

To date, little research has appraised water management guidelines. Some exceptions are Bennett (2009), who noted that apart from select recommendations within it, Australia’s National Water Quality Management Strategy guidelines (NWQMSG) had not been systematically reviewed since they were published in 1992. Bennett (2009) reviewed the history and objective of each recommendation in the NWQMSG, consulting over 100 stakeholders (Bennett 2009). Speaking to the value of participation in adaptive, integrated water management, they concluded that to “more accurately gauge the utility of the current suite of guidelines, and to inform any future guideline development, the perspectives of key stakeholder groups including the jurisdictions, water industry and local government would need to be better understood” (Bennett 2009, pg. 6). Higgins (2010) agrees calling for collaboration between authorities and actioning institutions to create standardised and relevant water management guidelines. Courtney et al. (2019) developed an assessment framework for FAO’s (2019) voluntary Small-Scale Fisheries (SSF) guidelines. Their framework organised that guideline’s 90 recommendations into 10 themes. Each recommendation was reworded to enable study respondents, made up of staff of USAID-funded fisheries project staff in the Phillippines and Indonesia, to rate the extent to which they felt each recommendation had been enacted in their respective working contexts (Courtney et al. 2019). Their assessment asked participants to rate guideline implementation status from 1 (low) to 5 (high), however provided little explanation as to the cause of success or failure of implementation (Courtney et al. 2019). A literature search for evaluations of guideline implementation showed these studies are again dominated by health sector examples. Fishway guidelines or guidelines relating to the motivations for fishway development such as food/nutrition security, poverty reduction, or fish species passing efficiency are rare or non-existent.

In summary, guidelines are published to guide a wide array of actions however there is no agreed standard form or quality assessment method. Literature regarding guideline creation processes and user assessments is mostly from the health sector and generally lacking. The current study aims to address this gap by evaluating three FFIGs, which represent a type of guideline that is currently proliferating and not well represented in the literature. Given the substantial resource investment that guideline production represents, this study contributes to guideline production and evaluation literature and fish-friendly irrigation system practice.

## Methods

This study has two components: document analysis of ADB (2020), Gregory et al. (2018), and FAO et al. (2020); and key informant interviews with guideline authors and end users. It was guided by the questions: *How are the FFIGs similar or different in structure and content?; To what extent are FFIGs perceived as useful by FFIG authors and users?;* and *What actions could improve FFIGs?*

### Document Analysis

Guidelines by Gregory et al. (2018); ADB (2020) and FAO et al. (2020) were selected due to their recency and the content similarity suggested by their titles. Understanding the unique value of each was of interest as the fourth FFIG published in five years is expected from MRC in 2023.

Thematic analysis (Weber 1990) of document content was conducted in MAXQDA qualitative data coding software (VERBI Software 2020). Document content analysis was used for systematic review of guideline content (Downe‐Wamboldt 1992). Guideline structure was compared with criteria developed by Nieuwlaat et al. (2021). First round thematic codes included: Challenge to be addressed; Role of fisheries; Recommendations; and guideline attributes. A second reading and in vivo (verbatim) coding was then conducted (Saldaña 2021). Coded text was sorted and consolidated. Coded segments were reviewed to confirm appropriate assignment to thematic headings and re-allocated as necessary. Themes were then compared across the three documents for consensus or dissonance.

### Interviews

Key informant interviews were conducted for insight into guideline formulation processes and perceptions of FFIG utility. Perception studies investigate individuals’ relative (Bernstein 1983) interpretations of social and physical contexts (Edwards et al. 2018). This lens was employed to illuminate how author and user experiences influence guideline utility and impact on practise.

Two rounds of semi-structured interviews were conducted. First round respondents were recruited by email and interviews took place between November 2021- January 2022. These included the 10 FFIG authors who agreed to be interviewed (of 14 total), 5 FFIG users (employed as irrigation engineers, fisheries, and rural development specialists) and one key informant (an author on Marsden et al. 2014) (Table 1). Two of the authors sampled had authored two reviewed guidelines. Of those two, one co-authored the present study. They were removed from the data analysis process to address that potential conflict of interest, contributing instead to this study’s conceptualisation, and editing. Users were recruited via snowball sampling (Handcock and Gile 2011), wherein authors recommended user respondents: either someone to whom they had given their FFIG; whom they knew had read it; or a target audience member they hoped was reading it. Participants were interviewed at a negotiated time via Zoom (Yuan 2011), for 30 min – 1 h.

**Table 1:** Overview of interview respondents

Interviewee reference code	GL author	GL user	Key informant
KI 1	Male, Australian	–	–
KI 2	Male, Australian	–	–
KI 3	–	Male, Australian	-
KI 4	Male, Australian	–	–
KI 5	Male, Canadian	–	–
KI 6	Male, Australian	–	–
KI 7	Male, English	–	–
KI 8	–	Male, New Zealander	–
KI 9	Male, English	–	–
KI 10	–	Male, New Zealander	–
KI 11	–	Male, Lao	–
KI 12	Male, English	–	–
KI 13	–	–	Male, Australian
KI 14	Male, English	–	–
KI 15	–	Male, Cambodian	–
KI 16	Female, Sri Lankan	–	–
KI 17		Male, Lao	–
KI 18	–	Male, Lao	–
KI 19	–	Female, Lao	–
KI 20	–	Male, Lao	–
KI 21	–	Male, Lao	–
KI 22	–	Female, English	–
KI 23		Male, Cambodian	
KI 24		Female, Cambodian	
KI 25		Male, Cambodian	
KI 26		Male, Cambodian	
KI 27		Male, Cambodian	

Second round interviews were conducted with an additional 11 participants in person between November – December 2022 in Vang Vieng, Lao People’s Democratic Republic (PDR), and Siem Reap, Cambodia at two fish passage master classes. Respondents were purposively sampled as FFIG end users. Respondents were irrigation engineers, fisheries biologists, irrigation department managers and representatives from local stakeholder organisations (Table 1). Second round interviews focused on addressing knowledge gaps emerging from the first round, and were conducted with assistance from local translators, for approximately 20 min each.

The interviews were recorded using Zoom or phone app and transcribed. These transcripts were reviewed for accuracy and then uploaded to MAXQDA and coded. Descriptive code headings were established in line with research questions. Content coded to each heading was reviewed, additional headings were created for emergent themes, then coded data was reviewed a third time and reallocated as appropriate.

## Results

### Document Analysis

Comparison with Nieuwlaat et al.’s (2021) guideline structure criteria showed Gregory et al. (2018) and ADB (2020) scored 9/22 respectively, while FAO et al. (2020) demonstrated 8/22 criteria (Table 2). FAO et al. (2020) did define the guideline target audience, guideline structure and objectives, however not in the format recommended by Nieuwlaat et al. (2021). FAO et al. (2020) contained no abstract. A recommendations summary, review mechanisms and impact evaluation were absent in all reviewed FFIGs.

**Table 2:** GL structural criteria

	Gregory et al. (2018)	ADB (2020)	FAO et al. (2020)
Title			
Identifies document as a GL	–	–	✓
Year of publication	✓	✓	–
Author information	✓	✓	✓
Abstract			
Objective	✓	✓	–
Target Audience	–	✓	–
Description of GL structure	✓	✓	–
Number of recommendations	–	–	–
Key recommendations	–	–	–
Summary of recommendations			
Summarised introduction	–	✓	–
Interpretation of strong recommendations	–	–	–
Interpretation of all GL recommendations	–	–	–
Justification	–	–	–
Main text			
Specific objectives	✓	–	✓
Description of problem	✓	✓	✓
Methodology of author selection and coordination	–	–	–
Funding	–	–	✓
Conflict of interest management	✓	✓	✓
Evidence review	✓	✓	✓
Document review process	–	–	✓
Comparison with recommendations of other GLs	✓	–	–
Limitations of GL development process	–	–	–
Plan for GL adaptation and updating	–	–	–
Total X/22	9	9	8

Adapted from Nieuwlaat et al. (2021)

FFIGs displayed different approaches to fish-friendly irrigation, demonstrated in the characterisation of challenges facing Mekong fisheries and solutions offered (Table 3). Gregory et al. (2018) took an ecological approach at irrigation system command area scale, explaining links between fisheries and irrigation in the introduction and briefly noting related social impacts and governance challenges. The guideline promoted watershed management that expands characterisation of water infrastructure impacts to include connected aquatic environments, and highlighted opportunities for fish in irrigated landscapes (Gregory et al. 2018).

**Table 3:** Summary of document content analysis showing similarities and differences between FFIGs

	Gregory et al. (2018)	ADB (2020)	FAO et al. (2020)
Framing	Physical impacts of WCI extend beyond the spatial area that is usually considered.	Current development investment approach to irrigation is harming fisheries.	Investments in technology are not enough to guaranteed improved livelihoods. Social factors must be accounted for.
Challenge characterisation	Impacts of WCI are not fully accounted for.	Challenges are characterised in economic terms including loss of livelihoods, out migration.	Challenges arise from lack of cross-sectoral collaboration in water resource management, lack of stakeholder representation, user conflicts, elite capture.
Approach	Opportunities for fish exist within irrigated landscapes.	WCI can be made more fish friendly.	WCI can be made fish-friendly and needs fish-friendly governance.
Recommended interventions	Extended Command Area assessment and Ecosystem Approach to Fisheries Management in Irrigation.	Barrier assessment and prioritisation; Fishways; Cost-benefit analysis via the Lower Mekong Basin Decision Support Tool.	Extend conceptualisation of WCI impact area; Stakeholder analysis; governance analysis; participatory approach; cross sectoral collaboration in WCI planning and operation.

FAO et al. (2020) brought the extended command area proposed by Gregory et al. (2018) forward, but in contrast focused strongly on social, participatory and governance aspects in its introduction and outline of opportunities for integrating fisheries into irrigated landscapes, noting this as a gap in other guidelines on this topic. More than the other guidelines reviewed, ADB (2020) focussed on economic factors. Fish friendly irrigation was contextualised through ADB’s Strategy 2030 and a novel cost-benefit analysis tool was presented aid decision-making. The stated target audience for each of the guidelines was FAO Fisheries and Aquaculture Department; ADB irrigation development staff; and water planners, water managers and civil engineers in general (Gregory et al. 2018; ADB, 2020; FAO et al. 2020 respectively).

FFIGs were presented in English only. All FFIGs contained advice for on-ground infrastructure engineers and high-level decision-makers but represented a complete guide for neither. Shiffman et al.’s (2004) executability and decidability criteria were not met; for example, FAO et al. (2020), Section 2, Step 1 - Part 2: Engaging Stakeholders, relates that late stakeholder engagement can hinder project success, but the optimal engagement timing is unspecified and not linked to water infrastructure project development timelines. Despite this, each guideline made useful and unique contributions fish-friendly irrigation practice, namely the spatial unit of analysis (extended command area approach – Gregory et al. 2018), a cost-benefit analysis tool (ADB 2020) and a stakeholder engagement framework (FAO et al. 2020).

### Respondent Perceptions

Author responses reflected guideline content. Author perceptions were more aligned across publishing organisations than reflected in guideline content. Guidelines were perceived as useful conceptual tools to introduce fish-friendly concepts to guideline users. In contrast to document analysis results, recommendations were convergent.

Author responses revealed an emergent narrative. A breakthrough occurred in 2018 after an observable increase in fish passage was demonstrated at Houay Peung wetland (managed by villagers at Pak Peung) in Lao PDR following fishway installation (KI 5). This generated interest in fish-friendly irrigation, notably from the Lao government (KI 2), but also the authors of Gregory et al. (2018), who invited a Houay Peung fishway project leader to contribute to their guideline, which was already being prepared (KI 12). Interest from ADB was simultaneously strengthened by the proof of concept demonstrated at Houay Peung. A rural development specialist at ADB, increasingly conscious irrigation’s negative impact on fisheries acknowledged a paradigm shift in ADB’s approach to multifunctional irrigation in the LMB through the production of ADB (2020). Finally, FAO et al. (2020) was produced by a collaborative team of researchers composed of staff from FAO, IWMI, WorldFish, and their partners, working together to address their own organisational foci as LMB multifunctional irrigation stakeholders. Some of the authors of FAO et al. (2020) had contributed to Gregory et al. (2018), their evolving experience having lead them from ecological and technical solutions to social drivers of development decision-making and a recentring of the development goals of fish-friendly irrigation in the LMB. Reflecting their own experience and professional commitments to improving nutrition security, enhancing livelihoods, alleviating poverty, as well as promoting fair, inclusive development and reducing conflict, they planned and produced the FAO et al. (2020) guideline.

The writing of ADB (2020) and Gregory et al. (2018) commenced with individuals who involved subject matter experts (for example economists, engineers) as the need for diversified content emerged (KI 1; KI 5; KI 12). In contrast, FAO et al. (2020) formulation more closely resembled Shekelle et al.’s (1999) five step process. An exception is that Step 5: *Review* was reported to be conducted by the publishing organisations rather than externally. An internal review was mentioned by ADB (2020) and FAO et al. (2020) authors, while none was mentioned in relation to Gregory et al. (2018). For all FFIGs, authors were chosen via professional networks (KI 6; KI 9; KI 12). Some had never met each other in person (KI 2).

Irrigation engineers and water managers were the most reported audience (KI 7; KI 8; KI 9; KI 14; KI 16). Others reported that development donors (KI 1; KI 2; KI 4; KI 6); universities (KI 4); or LMB national governments and departments (KI 1; KI 7; KI 8) were the target. All FAO et al. (2020) authors interviewed reported the same target audience, (irrigation engineers and water managers), which may reflect alignment fostered in that guideline’s inception workshop. In the absence of participation in the design and planning stages of ADB (2020) and Gregory et al. (2018), the lesser agreement between authors of those guidelines about target audience could be attributed to their contribution focused participation in the later stages of guideline production (Section 4.2.2).

Interview data showed the target audience was not involved in production of ADB (2020) or Gregory et al. (2018). Mixed responses were received from FAO et al. (2020) authors:

It is the first time we had brought together, like, the fisheries and irrigation folks, to come together and develop something from the get-go. […] In this case with a combination of FAO, WorldFish and IWMI kind of brought together to the same table these different, these different sectors. (KI 16)

I don’t think there was anybody actually […] To be honest with you, we should have had more people from the irrigation side. We didn’t have very many people from, if anybody, around from the irrigation side. (KI 7)

These responses imply development organisations were collaborating on this same topic of fish-friendly irrigation, however explicit input from the target audience (e.g. irrigation planners and managers from the target countries) is not described.

FFIGs aimed to bridge experiences between ecological, engineering and donor/investor sector operators (benefactors) and beneficiaries; with the goal of providing multi-functional, ecologically and socially sustainable irrigation development (KI 2; KI 6; KI 9; KI 14). As mentioned, for some authors guidelines signposted a paradigm shift in their approach to development (KI 5; KI 6). For another:

[We were trying to] basically show the value of the fisheries within irrigation. So that you demonstrate that the, the fisheries bring value, add value to the system, increase water productivity. (KI 7)

GL user perceptions differed slightly, reflecting a need for practical advice (KI 8; KI 10; KI 15; KI 18; KI 20):

The thing that we wanted to do was not argue the principle but say okay, how do you influence the design process and […] sit with them and say okay well, if you want to do this, we need to do it properly, what is the process to use to inform design? (KI 8)

Interviews revealed none of the reviewed guidelines were yet widely known or utilised by respondents. Specific guidance on fishway implementation was an assumed function of FFIGs (KI 11; KI 13; KI 15), however reviewed guidelines introduced fish-friendly irrigation and provided only general advice (KI 7) demonstrating dissonance in expectations of guideline function and utility. Users expressed demand for locally adapted guidelines (KI 11; KI 18; KI 19) and reported difficulty actualising recommendations (KI 10; KI 11; KI 15; KI 20). One user stated:

Most talk about the introduction of fish-friendly but it’s not enough. […] It doesn’t give enough information for evaluation. Needs more detail. (KI 20)

Authors reported awareness of guideline user demand for more specific, operational guidelines, however the disconnect between user expectations and guideline content resulted in the perception that FFIGs were incomplete. Despite this, most respondents agreed FFIGs represented a useful tool to initiate communication between disparate resource management sectors and governance levels (KI 1; KI 3; KI 5; KI 7; KI 11). This was noted as important due to the perception that resource managers:

They don’t really think beyond their remit or beyond their ministries remit. I mean it’s hard to get agriculture and fisheries people to talk together. (KI 12)

However, authors reported never having used their guidelines, or not for the purpose for which they were designed (KI 3; KI 5; KI 6).

Speaking more to the specificity of advice contained within the reviewed guidelines, challenges of writing to the appropriate scale were described by multiple authors (KI 3; KI 6; KI 14). The shortfall between FFIG content and specific measurements for detailed fishway design was recognised (KI 6), as was the destructive potential of inappropriately applying a blanket solution to diverse water management contexts (KI 10; KI 13). A solution was offered by one guideline user:

One of the challenges I see with guidelines when they’re produced at a sort of higher-level international space […] they’re very good at providing information […] but it doesn’t necessarily translate into outcomes on the ground without further work.

So the most successful way that we have found to actually produce on ground and in country outcomes is that we take those as broad scale large visionary type guidelines and then we use those almost as a teaching aid to introduce fish passage. And then we narrow that down into site specific. […] We do that in a way that it is experts supporting in-country partners to produce their own guidelines, so as opposed to an expert writing it from an international space. (KI 3)

The participatory, collaborative approach described by KI 3 somewhat addresses partitioned FFIG benefactor-beneficiary relationships and underlines that guidelines are not solutions in and of themselves, instead requiring further distillation with local experts into site specific operating procedures (Bennett 2009; Higgins 2010).

No evaluation mechanisms for any level (guideline, organisational, national or regional) were reported by authors (KI 9; KI 14) leading to uncertainty about FFIG impact on practise (KI 5; KI 12; KI 14; KI 16). One author explained:

We kind of envisaged what we’ve currently done with the guidelines. But what we haven’t done is maybe more significant, which is actually road-testing the guidelines. (KI 9)

Many authors didn’t know if their guidelines were being used (KI 4; KI 7; KI 12; KI 16). Authors reflected on a muted reaction, stating “it’s slower than we would have liked” (KI 12). It was observed that important networking opportunities had been hindered by COVID-19, significant as guideline authors reported conferences and conversations with key individuals were main dissemination strategies (KI 2; KI 6; KI 7; KI 9; KI 12; KI 16). Interview respondents demonstrated little awareness of reviewed FFIGs, with few exceptions (KI 3; KI 17). Some were aware of MRC guidelines not reviewed here (KI 11; KI 15; KI 21).

Speaking to factors that influence guideline impact on practice, authors reflected that foundational knowledge about fish passage is scarce (KI 7; KI 12; KI 13; KI 14). Factors inhibiting implementation of fish-friendly irrigation were that varied. First, FFIGs were only published in English language (KI 11; KI 15; KI 18). Although intentions to address this were reported (KI 16), or recognised (KI 6) by authors, no guideline users reported experience of translated guidelines. Others contended the approach and training of resource managers can make communication between sectors difficult (KI 1; KI 10; KI 14). The attitudes and experience of key stakeholders (KI 3; KI 8; KI 12), in particular irrigation engineers (KI 14) was discussed, as was succession after retirement of fish-friendly irrigation advocates (KI 5; KI 16; KI 22) and overcoming the legacy of development approaches that emphasise only irrigation productivity over environmental trade-offs and stakeholder consultation (KI 12; KI 15).

Social factors added complexity to fish-friendly irrigation uptake (KI 7; KI 22). Some authors noted power differentials between better resourced irrigation and the much smaller fisheries departments in LMB countries (KI 9). The same imbalance is reflected at local scales in the relative resource decision-making power or influence of fishers (often representing landless and other marginalised peoples) in relation to land-owners, and larger commercial agriculture businesses (KI 12; KI 14). Additionally, and difficulty quantifying socio-cultural values and benefits of fisheries was a challenge (KI 1). Fishway performance indicators such as economic and other evaluative measures are fledgling in the fish-friendly irrigation field (KI 1).

Political will and the role of law were underlined (KI 11; KI 14; KI 15; KI 18). Cross-sectoral collaboration was noted as vital and lacking (KI 1; KI 3; KI 12; KI 14), while the legal framework in which fish-friendly irrigation was nested was insufficiently detailed to ensure effective policy (KI 15). Users reported that FFIGs could be improved by the inclusion of local knowledge (KI 15) and recommended the inclusion of fish passage training in irrigation engineer training curricula, to pre-expose engineers to fish-friendly irrigation concepts (KI 11; KI 15; KI 18; KI 19; KI 20; KI 21).

## Discussion

This assessment documents that presently, water management stakeholders in the LMB are examining fish-friendly irrigation. They are investing in determining best practises, attempting to set practical standards for irrigated landscape multifunctionality by way of guidelines and exploring how to do this at the country (Lao PDR) and regional scales. This represents a paradigm shift to irrigation development in the LMB wherein diverse stakeholders are increasingly sensitive to fisheries and their benefits in irrigation systems, and are adopting approaches that reflect holistic development such as proposed by the SDGs. The unique contribution of each guideline (spatial analysis unit, cost-benefit tool, stakeholder participation framework – Gregory et al. 2018; ADB 2020 and FAO et al. 2020 respectively) may be strengthened by concerted application as part of an integrated management framework (Bouwer 2000).

This study demonstrated that FFIGs published by Gregory et al. (2018), ADB (2020) and FAO et al. (2020) shared similarities but also had fundamental differences. All three guidelines poorly reflected the structure and content criteria developed by Nieuwlaat et al. (2021). Although unsurprising as Nieuwlaat et al. (2021) was published after the reviewed guidelines, no alternative structural framework was reported by authors. All critically lacked a summary of clear and easily accessible recommendations (Table 2).

Similarly, the production process varied between reviewed guidelines. Perhaps due to select individuals within the author group driving design and planning, authors involved in the later stages of Gregory et al. (2018) and ADB (2020) were unclear about those guidelines’ stated target audience identity. For FAO et al. (2020), a design and planning workshop convened at the outset of that guideline’s production improved its authors’ ability to identify the stated target audience, however interviews revealed the breadth of this audience group (water planners, water managers and civil engineers) brought its own challenges in trying to address audience needs.

This was substantiated by user respondents who expressed vague advice as a hinderance to applying recommendations. No guideline structure or content evaluation mechanisms were detailed in the documents themselves or by interviewees which might have captured this.

Contrasts between guidelines were found in their content. Gregory et al. (2018) was a conceptual think piece showing how fish could be reincorporated into irrigated landscapes for ecological benefit. It was strengthened by the Houay Peung fishway case study, which was also foundational for ADB (2020). ADB (2020) sought to demonstrate fish-friendly irrigation in the LMB was economically viable, reflecting their organisational focus as a development donor/investor. FAO et al. (2020) drew on Gregory et al.’s (2018) extended command area conceptual framework and responded to the social challenges of fish-friendly irrigation, reflecting its’ publishing organisational foci. These differences demonstrate the positionality (Bourke 2014) of guideline producers (Table 3).

Five aspects emerged as limitations to the guidelines’ current form that improve utility and impact. These are: definition of target audience; engagement of target audience in guideline design; definition of guideline scope; specificity of recommendations; and evaluation. Interestingly these closely correspond with the five quality domains identified in a health sector guideline evaluation by Cluzeau (2003): scope and purpose; stakeholder involvement; development rigour; guideline clarity and presentation; and applicability.

By way of explanation, the stated goal of the reviewed guidelines was improving nutrition and livelihoods through fish-friendly irrigation in the LMB, however their intended audiences were vague, raising uncertainty about the professional representativeness of users sampled. Eccles et al. (2012) advise that although guidelines may be widely read, specifically defining the primary audience is useful to ensuring recommendations are fit for purpose. Due to the need for stakeholder inclusion to be balanced against production efficienc*y* (Cleaver 1999), the reviewed FFIGs can be interpreted as starting points (Nakayam and Fujikura 2006) from which local practises can be derived and developed with local stakeholders and knowledge. For example in their current form, the reviewed guidelines could function as conceptual introductions to fish-friendly irrigation or to inform water resource managers: firstly to increase conceptual awareness of multifunctional irrigation that considers fish in water infrastructure design, and secondly to integrate positional (Bourke 2014) differences between successive landscape management regimes (Bouwer 2000).

In the current study, despite the diverse disciplinary training of interviewed authors, irrigation experts were poorly represented, social and communication scientists were not engaged, and fish-friendly irrigation beneficiaries - local and national government departments, irrigation managers, fish and water resource users – were reportedly absent from the guideline production process (excepting country-based irrigation officials present in the FAO et al. [2020] design workshop). As with organisations, author experience and expertise informs individual positionality (Bourke 2014) which needs to be acknowledged to enable appropriately inclusive stakeholder engagement (Anggraeni et al. 2019) and combat bias (Shaneyfelt and Centor 2009). Siloed thinking was described by multiple guideline authors as a challenge to fisheries in the LMB. Literature sources such as Jackson (2012) extend siloes to include lacking dialogue between development organisations and beneficiaries, despite their observation that, although often separate, these two groups’ goals ultimately align. Authors interviewed agreed with literature sources (Markowska et al. 2020; Von Korff et al. 2012) that the meaningful engagement of diverse stakeholders at various hierarchical scales were required to create sustainable, mixed-use irrigation landscapes, however this did not occur in the production of the reviewed guidelines.

Related to questions of target audience identity and engagement are questions of scope. The tension between providing universal versus specific guidance revealed in interviews is discussed by Refsgaard et al. (2005), who state that as no universal water management context exists, designing effective and widely applicable guidelines is challenging. The reviewed guidelines presented general and more specific conceptual information about fish-friendly irrigation, for example various fishway types were illustrated in ADB (2020), and a step-by-step stakeholder engagement process is described in FAO et al. (2020). However all guidelines stop short of prescribing fishway designs, acknowledging that these are decisions that should be made by (or with extensive involvement of) local stakeholders, and that are highly dependent on local environmental, technical and social contexts. Clearly defining what functions a guideline does and does not perform may reduce user perceptions that guidelines are incomplete, and logically the opportunity to match target audience needs is greatest if the target audience is well defined and engaged. Key Informant 3’s assertion that the reviewed FFIGs need further distillation is supported in the literature. A study by Gagliardi and Brouwers (2015) found guideline impact was increased when accompanied with an implementation support tool, defined as a partner document that provides the specific operational information required to translate high-level guideline recommendations into practical action. This could be useful in the present context as locally specific partner manuals to the reviewed FFIGs as findings suggest that uptake of even poorly structured guidelines can be improved by implementation support tools as a feasible and widely applicable resource (Gagliardi and Brouwers 2015).

In the current study the same questions of scope and detail underpin user perceptions FFIGs are useful conceptually but insufficiently detailed to implement. Mindful of the influence of user perceptions of utility on guideline impact, user requirements should be central to FFIG production . Shiffman et al. (2005) and Refsgaard et al. (2005) agree that ambiguity in the suggested standard, amount, or strength of recommended actions can negatively impact implementation outcomes. To remedy this Refsgaard, Henriksen (2004), Bennett (2009) and Higgins (2010) underline that iterative discussions between guideline users and authors are needed to ensure guideline documents are congruent with user needs. Participatory approaches are supported in water management guideline literature (Bennett 2009) and can facilitate improved understanding of behavioural motivations and drivers of change (Nyam et al. 2020).

Finally, although importantly, none of the reviewed guidelines planned evaluation of their utility or impact. Evaluation is important to validate guideline quality (Azizi, 2020), while monitoring impact can aid in justifying investments in guideline production. One possible reason for a lack of evaluative measures is that guidelines are viewed as “end-of-pipe” solutions (Krywkow 2007), rather than blueprints or starting points (Nakayama and Fujikura 2006) for implementing practical change. Another likely reason is the difficulty in measuring benefits of fish-friendly irrigation and fishways, although various methods have been explored (e.g. Baumgartner et al. 2021b; Cooper et al. 2019; Dodd et al. 2018; O’Connor et al. 2022; Marsden and Stuart 2019). Due to their recency, it may be too early to accurately assess impacts of reviewed FFIGs, especially as dissemination was hindered by COVID-19. However, although optimal strategies for integrating guidelines into practise are not well understood (Shah et al. 2021), Nakayama and Fujikura (2006) support suggestions from key informants that guidance should be field-tested. Field-testing may facilitate FFIG improvement by revealing gaps in executability or decidability aspects of recommendations (Shiffman et al. 2005), or aspects that contribute to under-implementation (Callender 2018; Nakayama and Fujikura 2006).

## Conclusion

By reviewing three FFIGs published between 2018–2020, this study showed that while perceived as useful, attention to five key areas of guideline design could improve FFIGs to the benefit of authors and users. Specifically, these are: define guideline target audience; engage the target audience; define guideline scope; ensure recommendations are specific and actionable; and build guideline review and evaluation measures into guideline project lifecycles. Moreover, FFIGs should be available in local languages, although it is recognised that this is a costly and time-consuming process.

However, guidelines are not ‘end-of-pipe’ solutions (Krywkow 2007) in and of themselves. Instead their production reflects individual author attributes and training, those of the organisations they work for and the local, regional or global developmental context in which they operate. It is worthwhile acknowledging and interrogating these influences and in exploring how guidelines could be improved for greater impact and utility (Karppinen and Moe 2012). This study revealed social factors, such as prevailing governance structures, resource management department power disparities and practitioner training also impact uptake of fish-friendly irrigation. Including relevant training in water manager curricula may address this.

The interview respondent sample was modest and likely did not capture the full range of user perceptions or working contexts. Another limitation of this study was that it did not assess guideline impacts on the operational processes of the publishing organisations, noting only that the guidelines mark a paradigm shift in the development approach of those organisations. This could be an avenue for future research. Through the application of guideline structural criteria developed for the health sector, and qualitative attention to perceptions of guideline production and application by users, the present study contributes to water management, fish-friendly irrigation system development, scarce water management guideline evaluations and guideline evaluation literature more generally. Improving knowledge on guideline formulation may aid future authors construct clear and complete guidelines for enhanced utility and impact, ultimately supporting the incorporation of fish-friendly infrastructure into irrigated landscapes.

### Supplementary information

Supplementary Material
