# Supplementary material for: How Useful? Fish-Friendly Irrigation Guidelines for the Lower Mekong Lack Definition in Five Key Areas
Source: Environ Manage. 2023 Jul 15;73(1):102–14. doi: 10.1007/s00267-023-01855-4 (PMC10786998; doi:10.1007/s00267-023-01855-4)
Supplement: Supplementary file 1 — Supplementary Material [file 267_2023_1855_MOESM1_ESM.docx]

**Supplementary Material**

Table 4. Interview responses from FFIG authors and users on major themes relating to FFIS and FFIGs

|  | **ADB (2020) authors** | **FAO et al. (2020) authors** | **Gregory et al. (2018) authors** | **FFIG users** |
| --- | --- | --- | --- | --- |
| **Perceptions of fish passage technology status in LMB** | - A lot more awareness since 2018. - Fishways slowly gaining ground, especially in Lao PDR. - Big donors interested. - Capacity building initiatives commenced (e.g. master class). - Lots of talk about fish passage (Lao PDR). | - IWRM fairly fledgling. | - Fish passage very relevant to LMB. - Big donors interested. - Not yet on landscape scale implementation as desired. | - Fishery specialists becoming part of river development teams (Lao PDR). - New topic (Lao PDR) |
| **Target audience** | - ADB managers - Aid agency - Lao government - Charles Sturt University - ACIAR - Decision makers - The funder (ADB) - Villagers need to know how fish passage works. | - Irrigation engineers - Water planners and managers - Practitioners who are working in the area - Fisheries and irrigation sectors - Although not reported as the target audience, it seems there was a strategy to disseminate this to development donors i.e. World Bank and ADB. | - Not clear for FAO (Gregory) FFIG - Development organisations need to be aware - Line agencies (government departments) |  |
| **Was the target audience involved?** | - No although on ground works have involved target audience. | - Yes - No | - No | - No |
| **Challenges FFIG address** | - Raising awareness with major stakeholder of WCI trade-offs to inland fisheries. - Providing a 'legitimate' voice/evidence to natural resource managers in country to get FFIS included in new development projects/investments. - Provides successful examples of fishway installations. - Highlights the contribution that inland fisheries can make to achievement of SDGs. - Shows proof of concept. - Provides options for integrating fish into irrigation systems. - Addresses a knowledge gap about multifunctional WCI. | - Raising awareness with major stakeholder of WCI trade-offs to inland fisheries. - Provides successful examples of reincorporation of fisheries and community objectives into irrigation. - Highlights the contribution that inland fisheries can make to achievement of SDGs. - Promotes a collaborative approach to water management. - Addresses a knowledge gap about multifunctional WCI. | - Raising awareness with major stakeholder of WCI trade-offs to inland fisheries. - Provides examples of where and how fisheries can be reincorporated into irrigated landscapes. - Addresses a knowledge gap about multifunctional WCI. | - Raising awareness with major stakeholder of WCI trade-offs to inland fisheries. - Providing a 'legitimate' voice/evidence to natural resource managers in country to get FFIS included in new development projects/investments. - Highlights the contribution that inland fisheries can make to achievement of SDGs. - Addresses a knowledge gap about multifunctional WCI. |
| **How are GLs used?** | - Not in master classes. - We have GLs for other things that we do (e.g. running the master class) but these are internal documents. - it can and should be used more but not sure by whom or where. - At the high level GLs are well represented. - GLs are only part of the picture. Local people need to come up with localised solutions. | - I think they are useful and hope they are being used. - We have discussions underway with different organisations about how to try and use the GLs. - We haven't tested these. | - I've never learned anything from guidance documents. - GLs are only part of the picture. Local people need to come up with localised solutions. - No one brings them out in meetings. - This is a higher-level document. - At the time there were no other GLs for fish passage in the LMB. | - We hired an international consultant who uses GLs. - I use the MRC (2014) FFIG. - I use FFIG as an introduction to the concept. - GLs helped in the design of water infrastructure. - We found the GLs very useful. - GLs are only part of the picture. Local people need to come up with localised solutions. - Because of the GLs we have 5 fishways in the works. - Used as a tool to raise awareness. - Have been able to use the concepts. - I read the parts that are relevant to me. |
| **How did GL authors work together?** | - Spearheaded by an individual with experience in deploying development projects in LMB. - Knowledgeable others were invited to contribute. | - Write shop convened for the initial planning of FFIG. - Taken forward by engaged and committed contributors. | - Two lead researchers had been working on the concept for a while, invited a third to provide their practical expertise. | - N/A |
| **Writing process** | - GLs emerged as opportunity to create a lasting, hopefully positive, mark. - Writers contributed and sent to lead author for editing and submission to ADB. | - GLs were planned as part of a wider collaborative project between CGIAR centres and with FAO. | - Two authors had written the bulk of the document and invited another author to provide technical knowledge. | - N/A |
| **Dissemination** | - I would have held some events to try to disseminate it, but I'm retired now. - we go to conferences - Try to get in on existing investments. - Conference attendance prevented by COVID-19. - Web page. | - Trying to take this to the development banks to get funding. - Country level launches prevented by COVID-19. - Talks with influential stakeholders at conferences prevented by COVID-19. - Turned work from the same wide project into an infographic and decision support tool. (**Note:** I asked if the GLs were linked, he said he'd never thought more than a passing thought about that) - Funding coming to an end, there is a chance for this to be brought forward in new initiatives. | - Talks with influential stakeholders at conferences prevented by COVID-19. | - N/A |
| **Perceptions of when GLs should be used** | - Design phase. - Need to make sure this is right so that investment is not wasted. | - Design phase. | - Fish passage consideration ideally automatic for all new projects. | - Design phase. - Attach GLs to TOR for investments. - You need to consider the whole impact on your very first visit. - Need to make sure this is right so that investment is not wasted. |
| **What follow up  mechanisms are in place to measure GL take-up?** | - Not a lot of enthusiasm for monitoring. - Lots of work to be done on this. | - I'm afraid I didn't follow up (changed employers) so I assume others have followed up. - Not sure how you would measure impact. - Only way to measure impact would be to review new water infrastructure investments to see if their processes match the GLs. | - Not having a massive impact. - Lots of work to be done on this. | - N/A |
| **Are you aware of other  FFIGs?** | - I think FAO have one but I didn't study them. - Not aware. - Know about the forthcoming MRC FFIG. | - We checked about other GLs and found a gap, cannot recall which GLs but they should be mentioned **NB:** Gregory et al. (2018) GLs are referred to. | - If you've found several GLs they've evolved out of a single stream of research. - I know of some by Imperial College London about effects of aquaculture on fisheries in irrigated landscapes. | - Know about the new MRC ones. - Know of MRC (2014) FFIG. - I know of some but do not have access (language and content sharing barrier). - Not familiar. |
| **What do you perceive influences the effectiveness of GLs?** | - Personalities of people involved very important. - Got to be able to demonstrate results. - Lots of social work needs doing around the structure. - Legislative support. - Takes time to shift perceptions. - Should GLs be more specific? - No one size fits all approach. - Standardised approach. - Effective fishway siting. - Development needs to be locally appropriate. | - Got to be able to demonstrate results. - Lots of social work needs doing around the structure. - Need to target diffuse stakeholders. - Legislative support. | - Personalities of people involved very important. - Need to target diffuse stakeholders. - Legislative support. - Takes time to shift perceptions. - No one size fits all approach. - Standardised approach. - Effective fishway siting. - Mistakes are costly in $$ and mindsets. - We are not responding to a need; it's not widely appreciated. - If they are not read they cannot have impact. | - Personalities of people involved very important. - Got to be able to demonstrate results. - Lots of social work needs doing around the structure. - Need to target diffuse stakeholders. - Legislative support. - Should GLs be more specific? - No one size fits all approach. - Standardised approach. - Effective fishway siting. - Needs to be locally appropriate. - Mistakes are costly in $$ and mindsets. - Make it worthwhile for local people. - Takes time to shift perceptions. |
| **Recommendations (disaggregated)** | - FFIS consideration needs acceptance into national law. - Need more data to show proof of concept. - Requirement on the funder to consider/provide fish passage where appropriate in new developments. - Willingness of irrigation department. - Strict quality control standards and overseeing body. - Monitoring and evaluation of fish passages to learn, adapt and build evidence base. - Evaluation that is accessible to diverse water resource stakeholders. - Involvement of diverse stakeholders in WCI decision-making and design teams. - It takes time for GLs to get good, to build relationship and for the concept to take hold. | - FFIS consideration needs acceptance into national law. - willingness of irrigation dept (inclusion of fisheries unit in irrigation training?) - Balancing of budget allocation to support fisheries. - Involvement of diverse stakeholders in WCI decision-making and design teams. - Increase capacity of local people. - Monitoring and evaluation of fish passages to learn, adapt and build evidence base. | - FFIS consideration needs acceptance into national law. - Need more data to show proof of concept. - Willingness of irrigation department - Balancing of budget allocation to support fisheries. - More specific information about FFIS design and how to implement. - Monitoring and evaluation of fish passages to learn, adapt and build evidence base. - Involvement of diverse stakeholders in WCI decision-making and design teams. - Increase capacity of local people. - Translation of GLs into local languages. - It takes time for GLs to get good, to build relationship and for the concept to take hold. | - FFIS consideration needs acceptance into national law. - Need more data to show proof of concept. - Willingness of irrigation department. - Requirement on the funder to consider/provide fish passage where appropriate in new developments. - Balancing of budget allocation to support fisheries. - More specific information about FFIS and how to implement. - Strict quality control standards and overseeing body. - Monitoring and evaluation of fish passages to learn, adapt and build evidence base. - Involvement of diverse stakeholders in WCI decision-making and design teams. - Increase capacity of local people (training accessible in local language, during college to prime and upskill local workers). - Translation of GLs into local languages. - Collaboration space for fisheries and irrigation/ other water using sectors/ engineers and biologists. |
